# Exploring the potential for planning support systems to bridge the research-translation gap between public health and urban planning

**DOI:** 10.1186/s12942-021-00291-z

**Published:** 2021-08-18

**Authors:** Paula Hooper, Claire Boulange, Gustavo Arciniegas, Sarah Foster, Julian Bolleter, Chris Pettit

**Affiliations:** 1 Australian Urban Design Research Centre, School of Design, The University of Western Australia, Crawley, 6009 Perth, Western Australia; 2 KPMG Australia, Melbourne, Australia; 3 Geo-Col GIS and Collaborative Planning, Delft, The Netherlands; 4 Centre for Urban Research, RMIT University, Melbourne, VIC Australia; 5 City Futures Research Centre, UNSW, Sydney, Australia

**Keywords:** Planning support systems, Participatory planning, Scenario planning, Land use planning, Health, Health impact, Built environment, GIS

## Abstract

**Background:**

There is consensus that planning professionals need clearer guidance on the features that are likely to produce optimal community-wide health benefits. However, much of this evidence resides in academic literature and not in tools accessible to the diverse group of professionals shaping our cities. Incorporating health-related metrics into the planning support systems (PSS) provides an opportunity to apply empirical evidence on built environment relationships with health-related outcomes to inform real-world land use and transportation planning decisions. This paper explores the role of planning support systems (PSS) to facilitate the translation and application of health evidence into urban planning and design practices to create healthy, liveable communities.

**Methods:**

A review of PSS software and a literature review of studies featuring a PSS modelling built environmental features and health impact assessment for designing and creating healthy urban areas was undertaken. Customising existing software, a health impact PSS (the Urban Health Check) was then piloted with a real-world planning application to evaluate the usefulness and benefits of a health impact PSS for demonstrating and communicating potential health impacts of design scenarios in planning practice.

**Results:**

Eleven PSS software applications were identified, of which three were identified as having the capability to undertake health impact analyses. Three studies met the inclusion criteria of presenting a planning support system customised to support health impact assessment with health impacts modelled or estimated due to changes to the built environment. Evaluation results indicated the Urban Health Check PSS helped in four key areas: visualisation of how the neighbourhood would change in response to a proposed plan; understanding how a plan could benefit the community; Communicate and improve understanding health of planning and design decisions that positively impact health outcomes.

**Conclusions:**

The use of health-impact PSS have the potential to be transformative for the translation and application of health evidence into planning policy and practice, providing those responsible for the policy and practice of designing and creating our communities with access to quantifiable, evidence-based information about how their decisions might impact community health.

## Background

A significant public health challenge of the 21st century is the rising rate of non-communicable diseases, particularly declining rates of physical activity and mental health and increasing prevalence of obesity, diabetes and cardiovascular disease. These are significant contributors to Australian and worldwide morbidity and chronic disease burdens [1, 2]. The design of the built environment is integral to encouraging positive health and well-being behaviours. The past decade has seen a proliferation of research documenting the impact and associations of the built environment’s many design features that support or undermine residents’ public health behaviours and outcomes [3–5].

Studies exploring relationships between the built environment and health behaviours and outcomes [6] have benefited from the emergence and application of Geographic Information Systems (GIS) tools and technology [7] that have been described as a significant innovation of social science research. GIS offers the opportunity to integrate spatial information from a range of sources into a single framework, and to use these data to develop precise quantitative measures of the built environment [8]. This has enabled a new generation of environmental exposure measures including walkability indices (and use mix, residential density, street connectivity); access (distance) and travel times to daily destinations, food outlets and parks; and the amount of greenspace [8]) that have been essential to explain and quantify the associations between features of the built environment and a range of health and wellbeing behaviours and outcomes such as walking, physical activity, food purchasing behaviours and weight status, mental health and sense of community [6, 8, 9].

### The public health-urban planning research-practice gap

Despite the mutual historical origins of urban planning and public health [10], by the late 20th century, the two disciplines have largely come to function as disconnected domains of knowledge and action [10]. Moreover, whilst the translation of public health research into tangible health benefits via modifications of urban planning policy and practice is a key intended outcome of these research endeavours [11], a research-translation gap remains between the ambitions of public health and the planning for, and delivery of, healthy, active communities [12]. It is essential to understand why this gap remains and how it can be overcome? One potential explanation relates to the type of health-related evidence (or science) needed by planners and planning policymakers for it to be utilised and applied [9]. Planning professionals need clearer guidance on the features that are likely to produce the optimal population health impacts [11, 13, 14]. However, several authors have indicated that public health evidence and the spatial measures of the built environment rarely provides quantifiable, evidence-based information about the potential health impacts of urban planning policies and decisions [13, 14] or match the interactive and participatory nature of planning decision making [15]. Moreover, a lot of this evidence resides in academic literature and not in tools accessible to the diverse group of professionals shaping our cities [10, 16].

This situation prompts us to consider how we can turn health research or science and data into meaningful information to foster collaboration between researchers and planning professionals? [12]. As such, there is a need to identify and test innovative digital tools that might help support the translation and application of health evidence into urban planning and design practices to create healthy, liveable communities.

### Planning support systems for scenario planning: a tool to bridge the research-translation gap?

Planning support systems (PSS) are spatial, data-driven, computer-based tools that integrate GIS and decision support functionality to convert data into meaningful information to support the activities of planning professionals [18, 19]. They typically consist of three critical components: spatial data, models and geo-visualisation [15] that allow for the dynamic simulation, testing and evaluation of different urban development proposals [18, 20, 21]. They contain dynamic maps supported by interactive interfaces to allow sketching and editing of spatial layers within the GIS with real-time indicators [17]. Moreover, PSS can be combined with custom-built models to link outcomes of interest to the spatial data layers to allow for the dynamic evaluation of the potential impacts of design proposals on health and well-being outcomes [18, 19].

PPS have been used as analytical tools for land use and transportation planners to test and evaluate travel, environmental, and economic impacts of different development or redevelopment scenarios and receive feedback about the implications of those assumptions. Incorporating health-related metrics into the PSS’s provides an opportunity to apply empirical evidence on built environment relationships with health-related outcomes to inform real-world land use and transportation planning decisions [22] enhance planning professions understanding of how different scenarios or approaches to neighbourhood design might impact community health and well-being and assist them in making informed and evidence-based planning decisions and practices? This raises the questions, could PSS with health impact models contribute to bridging the policy-relevant research – evidence-based policy and practice gap?

While technological advances have seen a rise in PSS development supporting planning processes and practices [23], there remains a lack of digitally enabled collaborative tools to facilitate health-oriented urban planning. PSS have not been widely studied in the context of integrating health evidence or promoting health impact assessments, bringing ‘health’ front and foremost in the design and planning processes. We hypothesise that PSS represent a potential toolkit for the translation and application of health evidence into urban design and planning practice by estimating impacts and communicating outcomes related to urban planning, as well as explicitly linking empirical health evidence to allow for the modelling and estimation of potential population health impacts of design scenarios or proposals [24].

There have been several reviews focussed on the development and progress of PSS [18, 19, 21, 25–28] and the sectors or fields of application (e.g., land use planning, water management, climate change adaptation) that PSS have been developed for and used in within the domain of spatial planning [29]. This paper addresses a gap in the PSS literature concerning the role of PSS to facilitate the translation of health evidence, focusing on the application of PSS within the context of a health impact application. Additionally, previous reviews into the use of PSS in the planning profession have concluded that PSS research still has to prove its added value to planning practice [28] through empirical research that moves away from experimental case studies towards real-world planning problems [30–33]. A better understanding of the benefits and usefulness of PSS, and specifically health impact PSS, for improving planning processes, and professional’s willingness to use them, are thus needed to advance the field further. As such, this paper also presents a case study of an application of a pilot health-impact PSS, informed by the literature review findings, to a real-world planning situation to evaluate its usefulness and benefits [27] in demonstrating and communicating potential health impacts of design scenarios.

The objectives of this paper are to:

1. Conduct an inventory of existing PSS software that would allow for the integration of an evidence-based health impact assessment component;
2. Conduct a literature review to identify studies featuring a PSS with a built environment and health impact assessment for designing and creating healthy urban areas, neighbourhoods and communities;
3. Evaluate the use of an empirically-based health impact planning support system (the “Urban Health Check”) to a real-world urban infill development scenario to assess how a PSS modelling health considerations can be used to educate planners and communities on the potential health impacts of their decisions.

We present the health impact PSS review methods and results, followed by a case study approach to outlining the development, application, and evaluation of the Urban Health Check.

## Methods

### Inclusion and exclusion criteria

The peer-reviewed papers’ inclusion criteria were: (1) original journal articles of published, peer-reviewed, empirical studies; (2) in the English language and (3) published since 1995. The papers’ focus needed to have a PSS with an interactive and analytical component focussed on the built environment, plus a health outcome/s being modelled or estimated. Surveillance or map visualisation systems that provided a portal for the presentation and overlay of spatial data layers were not the focus of this paper (i.e., census population/health data overlaid with the built environment’s spatial datasets). Likewise, the broader domain of planning support systems that provide a further departure from the built environment was not included in this review.

For the current examination, a relatively broad definition of the “built environment” was used to identify and understand the range of modifiable factors in the external local environment (i.e. the neighbourhood, urban area or city) that may impact people’s health behaviours (i.e., physical activity) or outcomes and had been modelled as part of a PSS. Likewise, a broad approach was taken to defining “health outcomes” for this review—encompassing all types and dimensions of self-reported or objectively assessed physical activity or any health behaviour or outcome plausibly hypothesised (and supported by existing literature) to be impacted by built environments. These include, for example, walking (recreational / transport walking), moderate-to-vigorous physical activity, obesity, public transport use.

### Search strategy

We selected all PSS studies in which software was used as the main component of the PSS. Other more generic software tools were identified utilising web-based scientific search engines, previous reviews [18, 19, 21, 25–28], electronic libraries and databases, and personal networking and knowledge. Citations of studies featuring PSS tools were retrieved through a series of searches in the PubMed, Web of Science, Scopus, Elsevier Science Direct, and Ingenta Connect databases. Searches were conducted using combinations of keywords within the title and abstract based on GIS implementations under the GIS and Society research area’s umbrella. Table 1 outlines the health- and planning support system-related search terms that were used. Database searches were supplemented with citations retrieved manually from relevant papers and reviews. We also identified and obtained four previous review papers on PSS use from the planning, geospatial and computer science fields [18, 19, 21, 23, 26].

**Table 1:** Health and planning support system related search terms

Health-related search terms	Planning support system search terms
Health Health impact Health impact assessment Health assessment Health outcomes Collaborative health planning Healthy cities Liveable cities	Planning support systems Spatial planning support Spatial decision support Scenario planning tools Scenario planning software Spatial planning decisions Geomodelling tools Geodesign Participatory GIS Public participation GIS Group spatial decision support systems Collaborative GIS Dynamic GIS Geocollaboration Communityviz Maptable Interactive table Touch table

### Study selection and data extraction

The titles and abstracts of all retrieved citations were screened by three reviewers (author one, two and three of the present article) and examined for their eligibility and mention of words meeting the search criteria (i.e., health, PSS). Duplicates were manually removed, and the full-text articles of included abstracts were retrieved and further screened by the same three reviewers to determine final eligibility. Discrepancies between reviewers surrounding a particular study’s eligibility were resolved by further evaluation and consensus amongst all authors. Information was extracted from each paper on the PSS tools and identified software relating to the following thirteen criteria (Table 2) and collated into a results matrix/table for analysis.

**Table 2:** Data extraction criteria

1	Tool’s name and the city and country in which it was developed and applied
2	Software/hardware characteristics—proprietary/license based or open-source; cost; online/cloud-based and/or offline, desktop, smartphone app, interactive table; others; distributed or face-to-face.
3	Functions and use—communication; spatial data visualisation; pre-built analysis modules; sketch planning and editing of spatial data layers; 3D visualisation; health impact analysis/modelling; individual or group decision making.
4	The user interface/information mediums—maps, graphs, charts, reports
5	The scale of the project/scenario being applied to: precinct/neighbourhood/city/metro/region/country
6	The target application/planning-related task or stage of a project cycle or decision process the PSS intended to support (site and context analysis, concept design, community consultation, design review, design documentation)
7	The intended users—who are the PSS intended for/target audience—planners/policymakers/community/elected members, others
8	The built environment/urban design exposure features have been used—e.g., density, land use mix, street connectivity, public open space, others
9	The health-supportive behaviour or outcomes impacts that have been used/estimated for the health impact scenario mode—e.g., walking, physical activity, mental health
10	The source of health data/evidence used
11	The scale at which the of health outcome data is collected/modelled—individual or geographic unit/s
12	The predictive/statistical modelling technique was used to estimate the health impacts
13	The population demographics the health impact is estimated for—children, young adults, adults, and older adults

## Results

### Review of PSS software

Eleven PSS software applications were identified (Table 3). Of these, three were identified as having the capability to undertake health impact analyses: (1) CommunityViz (http://communityviz.com/); (2) Urban Footprint (http://urbanfootprint.com/); and (3) Envision Tomorrow (http://envisiontomorrow.org/). Developed as an ArcGIS^®^ extension, CommunityViz allows for customisable programming of outcomes of interest (i.e., health behaviours). The CommunityViz Scenario 360 software transforms a GIS into a dynamic system that allows co-sketching and rapid editing of the spatial layers. The attributes of the geographic features are driven by formulas that update automatically as the user makes changes. Each time a spatial feature on the map is changed (i.e., added, deleted, moved, its attributes changed) by the user, the system is updated, the formulas automatically re-run, and the outcome indicators dynamically changed.

**Table 3:** Inventory of planning support system software

PSS Software	
CommunityViz	https://communityviz.city-explained.com/ (36)
Envision Tomorrow	http://envisiontomorrow.org/
Online What If?	https://aurin.org.au/resources/decision-support/what-if/ (37, 38)
UrbanFootprint	https://urbanfootprint.com/
ESRI GeoPlanner	https://www.esri.com/en-us/arcgis/products/arcgis-geoplanner/overview
ESRI City Engine	https://www.esri.com/en-us/arcgis/products/arcgis-cityengine/overview
TNO Urban Strategy	https://www.tno.nl/en/focus-areas/traffic-transport/roadmaps/smart-and-safe-traffic-and-transport/societal-impact-for-accessibility-and-liveability/big-data-ecosystems-collaborating-on-data-controlled-cities/urban-strategy-brings-planning-effects-into-clear-focus/
UrbanSim/UrbanCanvas	https://urbansim.com/ https://urbansim.com/urbancanvas
Healthy Urban Route Planner	https://www.ams-institute.org/urban-challenges/resilient-cities/healthy-urban-route-planner/
Tygron Geodesign Platform	https://www.tygron.com/nl/
INDEX Plan Builder SPARC	http://www.crit.com/sparc/

### Review of health impact PSS studies

A total of 243 potential articles were retrieved from the search. Initial screening on the title and abstract identified 39 articles that met the eligibility and inclusion criteria and for which the full-text articles were retrieved for review. Of these, only three papers that met the inclusion criteria of presenting a PSS customised to support health impact assessment with health impacts modelled or estimated due to changes to the built environment were therefore included and described below. The full results against the thirteen extraction criteria for these studies are outlined in Table 4.

**Table 4:** Health impact planning support systems

Paper	Schoner et al. [39] Bringing health into transportation and land use scenario planning: Creating a National Public Health Assessment Model (N-PHAM)	Boulange et al. [40] Improving planning analysis and decision making: The development and application of a Walkability Planning Support System	Ulmer et al. [24] Application of an evidence-based tool to evaluate health impacts of changes to the built environment
Health PSS tool/system	National Public Health Assessment Model (N-PHAM)	Walkability Planning Support System	Coalitions Linking Action and Science for Prevention (CLASP)
Country	USA	Australia	Canada
Description	Provides baseline built and natural environmental conditions and pre-calculated health outcomes/levels Health Module “engine” contains computed equations describing the association between built environment features and health outcomes N-PHAM calculates/forecasts new outcome values based on the provided custom inputs/user changes to the built and natural environmental measures	Simulates changes in the built environment and modelling the impact these would have on transport walking behaviours: Measure the walkability of an area Test potential impacts of future policies and scenarios by allowing users to create and manipulate a virtual representation of an urban precinct Assess selected health impacts of these planning decisions/scenario for the community	Designed to predict physical activity levels, health-related indicators and GHG emissions associated with proposed land use and transportation developments
Software and hardware characteristics	Web-based API plug-in Integrates with multiple existing scenario planning platforms and software applications, allowing users of available scenario planning tools (CommunityViz, Envision Tomorrow, UrbanFootprint) to choose an area of interest represented by Census block groups which return baseline input and outcome values for each block group, as well as aggregated values for the study area These data are then available to the tool user to map and analyse data in ways specific to the respective tool	ArcGIS + CommunityViz 5.1 Commercially available software package owned and administered by City Explained Inc It is customisable and is an extension of ESRI’s ArcGIS Displayed on a touch-enabled 46-inch MapTable that can support up to 10 people around its screen	ArcGIS + CommunityViz 5.1 Commercially available software package owned and administered by City Explained Inc It is customisable and is an extension of ESRI's ArcGIS
Functions and user interface	Spatial data visualisation Dynamic interface for sketch-planning + editing of spatial layers Maps, charts Health impact analysis and modelling	Spatial data visualisation Dynamic interface for sketch-planning + editing of spatial layers Maps, charts Real-time Health impact analysis and modelling	Spatial data visualisation Dynamic interface for sketch-planning + editing of spatial layers Maps, charts Real-time Health impact analysis and modelling
Urban magnitude/scale of the project/scenario application/s	Precinct Census block groups Region/state National	Precinct Suburb	Precinct Suburb Postal codes
Built environmental/urban design layers	Gross population density Gross employment density Jobs within a 45-min transit commute, distance decay, walk network and GTFS schedule travel time Employment entropy index using a 5-tier employment classification scheme Retail jobs within a 5-tier employment classification scheme % of CBG employment within 1/4 mile of a fixed guideway transit stop Network density—facility miles of pedestrian-orientated links per square mile Street intersection density, weighted auto-orientated intersections eliminated % of land cover developed as open space % of land area covered by tree canopy % land cover = forest % land cover = natural % of land cover = developed open space or natural space	Land use mix (commercial, education, industrial, parkland, residential) Dwelling density Housing diversity score Local living destination score—convenience (convenience store, newsagent or petrol station); supermarket; speciality food destination (fruit and vegetable, meat, fish or poultry store); post office; bank; pharmacy; general practice/medical centre; dentist; community centre; childcare facility; library; Closest train station (< / > 800 m); Closest bus stop (< / > 400 m) Street network—intersection density	Length of roads; bicycle and sidewalk facilities; Distance to nearest major arterial, school and transit stop/station; Accessibility to major regional destinations; several density vectors, including net-residential, intersection, schools, transit stop and type of each food location (sit down and fast food, grocery and convenience stores); Land use—an entropy-based measure of the mix, retail floor-to-land area and park area
Health behaviour or outcomes or impacts have been used/estimated as coefficients for the health impact scenario model	Transportation walking (binary participation + continuous duration) Leisure walking (binary participation + continuous duration) Transportation biking (binary participation + continuous duration) Auto travel/sedentary time (binary participation + continuous duration) Recreational physical activity (binary participation + continuous duration) Body mass index (continuous) Overweight (binary) Obese (binary) Kessler-6 mental health—moderate (binary) Fair or poor general health (binary)	Transportation walking	Walking and biking for exercise; Walking and biking to work/school Body mass index Daily energy expenditure Blood pressure; Walk/bike trips/day, Transit trips/day, Automobile trips/day, Kilometres of travel/day Estimated vehicular emissions of CO_2_/day
Scale at which the health outcome data is collected/modelled	Census block	Meshblock (the smallest geographic region on the Australian Statistical Geography Standard)	Postal code
What predictive/statistical modelling technique was used to estimate the health impacts?	Likelihood of participating in the activity = binary health outcomes = binary logistic regression	Multi-level, multivariate logistic regression To estimate the probability that an individual participates in transport-walking, the formula takes in the values for each built environmental variable multiplied by the corresponding regression coefficients and summed with the constants	Multivariate regression models were used to predict the value of each health outcome/behaviour based on each participant's built environment and demographic/socioeconomic characteristics Four different types of regression model were used, depending on the type and distribution of the outcome variable: linear, log-linear, binary logistic and two-stage (zero-inflated). In each case, a base model was first built to include any statistically significant (p < 0.05) demographic/socioeconomic variables
Population demographic/s	Adults 18–64 Older adults 65 +	Adults > 18 years	Adults > 18 years
Target application/planning-related task or stage	Baseline analysis Scenario testing	Baseline analysis Scenario testing	Baseline analysis Scenario testing
Intended users	Planners Community Policy makers	Planners Community Policy makers	Planners Academics Policymakers
Does it support individual or group decision making?	Individual Group	Individual Group	Individual Group

- In the United States, Schoner et al. [39] developed the “National Public Health Assessment Model” (N-PHAM) that included a Health Module “engine”, which contains a set of equations describing the association between built environment features and health outcomes, and a database of nationally available baseline input data and pre-calculated baseline health outcomes. This builds on the regional and state-based modules initially created in the Urban Footprint and Envision Tomorrow tools to develop a consistent, nationally applicable decision-support planning tool to quantify the health impacts of changes to the built and natural environment. NPHAM includes baseline conditions at U.S. Census block groups for the entire United States. Users can create custom inputs based on a future scenario of the built, natural and social environments. These environmental changes are used to calculate new values for the predicted health outcomes.
- In Australia, Boulange et al. [40] designed the Walkability Planning Support System using a commercial software platform/extension to Esri’s ArcGIS: CommunityViz Scenario 360, an extension to Esri ArcGIS, and linked with a MapTable. This was applied at a suburb scale to test different urban renewal scenarios. The tool features include (i) automated calculation of built environment variables; (ii) “sketch planning” functionality; and (iii) suite of indicators including a walkability indicator that estimates the probability that an adult would walk for transport. This tool was implemented to determine walkability indexes for three suburbs in Victoria, Australia. The regression coefficients used in the underpinning formula were informed by multivariate logistic regression analysis of a large-scale transport and activity study that controlled for socio-demographic factors associated with transport walking.
- In Canada, Ulmer et al. [24] designed an empirically based health and greenhouse gas (GHG) impact assessment tool linking detailed walkability and regional accessibility measures with travel, physical activity, health indicators, and GHG emissions. Built environment measures were correlated with health and demographic characteristics from the Canadian Community Health Survey and travel behaviour from the Transportation Tomorrow Survey. Results were incorporated into an existing software tool and used to predict health-related indicators and GHG emissions for the Toronto West Don Lands Redevelopment.

Several other tools were identified that model or provide indicators of built environments conducive to positive health outcomes based on health research. For example, the Walkability Index Tool as part of the Australian Urban Research Infrastructure Network (AURIN) [11] measures the walkability of user-specified areas (i.e. suburb, Australian Bureau of Statistics (ABS) Statistical Areas and user‐specified road network buffers) for any Australian urban area (https://aurin.org.au/resources/workbench-user-guides/portal-user-guides/analysing-your-data/walkability-tools/). Similarly, the PedCatch tool (http://pedcatch.com/) is an open-source simple agent-based walkable catchment tool that researchers can use, urban designers, planners, and policymakers to test proposals for improving walkable neighbourhood catchments [42]. However, while both are founded on empirical health research, they do not model potential health impacts due to changes to pedestrian catchments’ walkability and thus were excluded from this review.

### Case study: development and testing of an evidence-based health impact planning support system

The Western Australian Government’s land development agency, DevelopmentWA, were preparing a redevelopment strategy for a former high school site in metropolitan Perth, Western Australia. They were keen to embed the consideration of health outcomes in their community consultation and decision-making processes into the planning and design of the site using locally relevant research evidence These conditions motivated the development of a health impact-based planning support system (“The Urban Health Check” PSS), informed by the literature and software review, that was capable of simulating changes in the built environment and measuring the impact these changes would have on the community’s surrounding neighbourhood and potential health behaviours.

This also provided a unique opportunity to evaluate the usefulness and benefit of applying a health-impact based PSS to a real world scenario to inform planners and communities on the potential health impacts of their decisions. The aim of this case study is to report on the results of this evaluation.

### Study site

The redevelopment site was a former high school and associated grounds, including a sports oval/ playing field totalling 11.9 ha in area. This was located in the suburb of Hamilton Hill, approximately 17 km south west of the Perth central business district. The area of focus for this PSS was the high school redevelopment site, plus the surrounding community within an 800 m buffer. This distance was chosen as it has been associated with transport walking measures such as walking trips and physical activity [43] and was the focus of the community consultation.

Area around the school was typical suburban area. Predominantly (71 %) low density detached residential dwellings of which three quarters (73 %) had three or more bedrooms. Less than 6 % of dwellings within the suburb are apartments. There are currently no mixed use or retail or public open space within a 400 m walkable catchment of the re-development site. The suburb of Hamilton Hill.

### Development of the Urban Health Check PSS

**Fig. 1:**
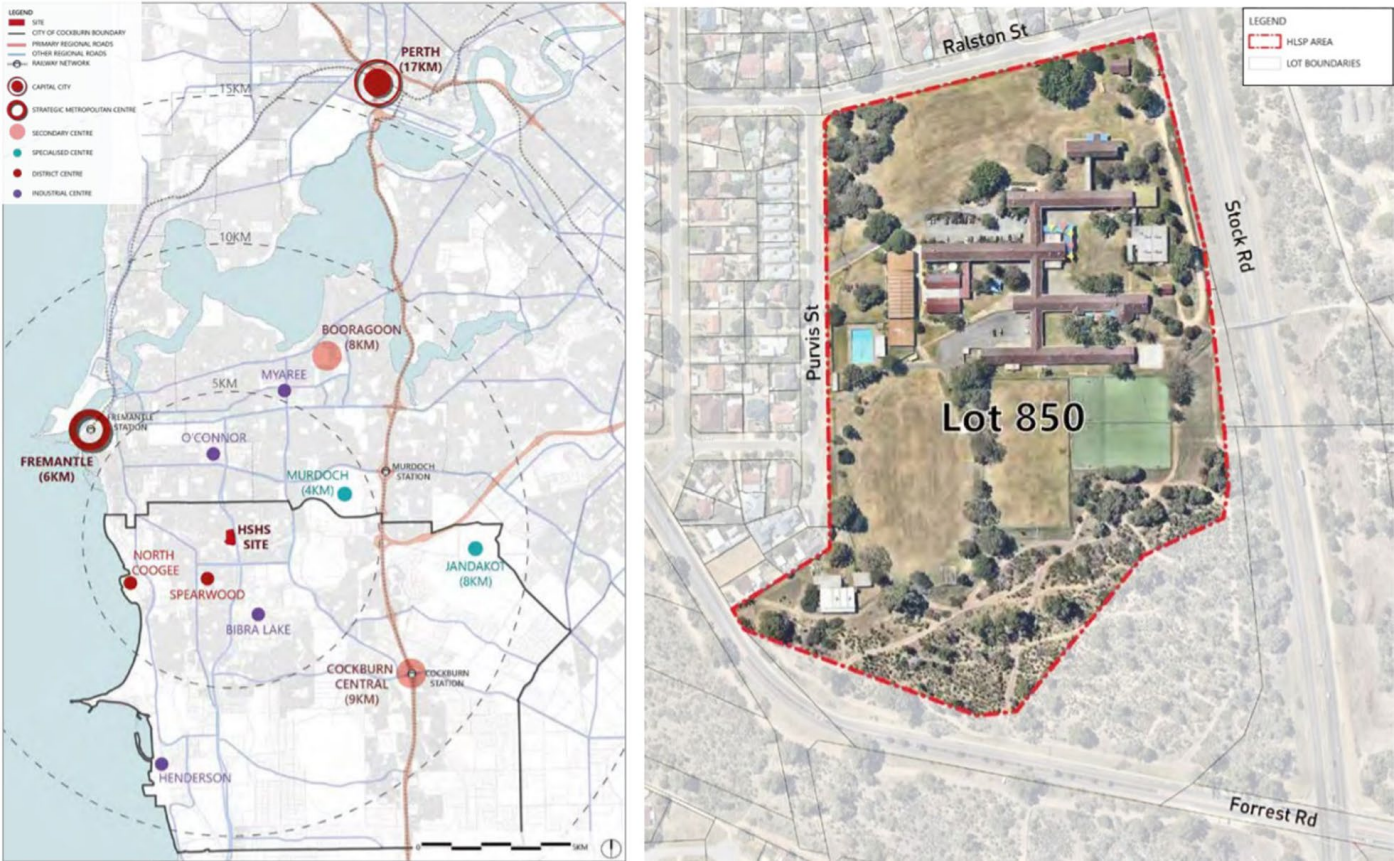
Site location. Source: Development WA: Hamilton Senior High School Local Structure Plan  [44]

The Urban Health Check was developed using *CommunityViz Scenario 360* software [36]. This was selected as it enabled customised programming of our outcomes of interest with local spatial data and locally-derived health evidence as an extension of ESRI’s ArcGIS. Seven spatial indicators were developed to assess the built environmental performance of different concept plans developed for the site: (1) Housing diversity (lot sizes); (2) amount (number and area) of parks; (3) access to public transport; (4) access to parks; (5) access to playgrounds; (6) access to sports parks; and (7) access to destinations. The indicators were chosen as they addressed issues previously raised by the community and were features known to be associated with health behaviours [2, 9]. Dynamic charts were created to represent the values of these spatial indicators (see Fig. 1).

The spatial indicators were linked to formulas to indicate potential health impacts. Earlier work from the RESIDential Environments (RESIDE) project had estimated the relationships between the built environment’s multiple features and a range of health-supportive behaviours and well-being outcomes for participants living across Perth, Western Australia [45]. A formula was programmed into the CommunityViz to estimate the probability that an individual undertakes (any) transport walking that took in the values for each spatial indicator multiplied by the identified coefficients. A baseline probability of walking was computed for the current condition at the case study site, and dynamic charts were programmed to represent the estimated values that automatically updated when alternative design options were sketched and changes were made to the spatial layers.

### Applying the Urban Health Check PSS-design review and community consultation

Three concept plans developed by a planning consultant were modelled in the Urban Health Check PSS. The spatial indicators quantified the changes in built form and access from each residential lot (I.e., the surrounding community) that might result from the different design concepts. The metrics derived for each concept were provided to the planning consultant to refine successive plans. The Urban Health Check PSS was also used as an activity during a three-hour traditional (drop-in) townhall-style community engagement and consultation event for the redevelopment project. The PSS was presented on a large (46 in.) touch-enabled MapTable [46] that was large enough to accommodate a group of 10 people and provides an interactive environment to support community engagement and planning participation around the PSS. Community participants viewed the study area’s baseline spatial layers and metrics. Participants were then invited to sketch into the system the proposed concept plan for the site, and instant feedback was given against each of the spatial (n = 7) and health (i.e., walking) indicators. Participants were then encouraged to sketch in alternative design ideas (i.e., edit, delete or add features) and got instant feedback against the same indicators. A facilitator helped users navigate the interface, explained the spatial and health indicators, and helped interpret the results.

**Fig. 2:**
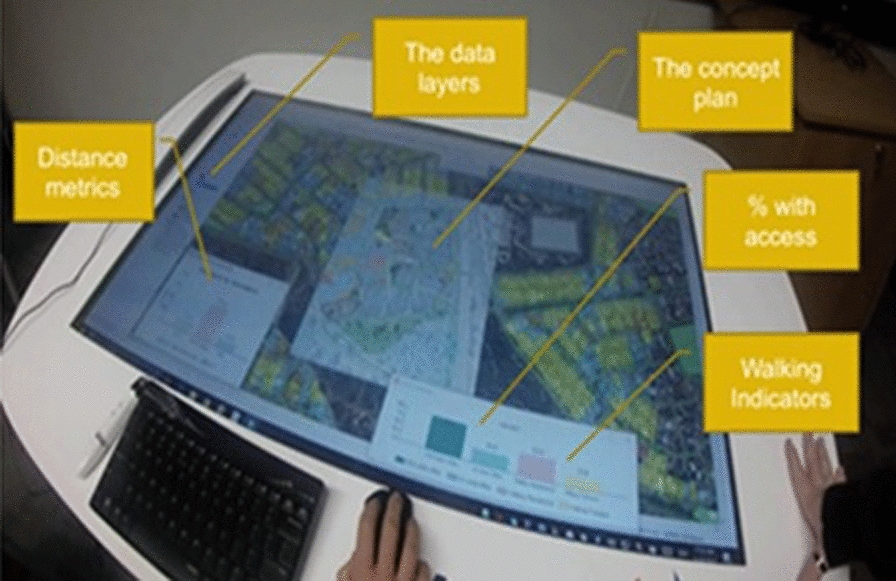
PSS interface, developed using CommunityViz, used at the community consultation

Figure 2 illustrates the user interface of the CommunityViz displayed on the MapTable. On the left side, the table of contents lists the spatial layers which can be manipulated using the ‘sketch tools (e.g., users can add new points, resize polygons or change attributes in features). As soon as the spatial layers were edited, the dynamic indicator charts displaying the spatial indicators’ values and estimated walking outcomes were automatically updated.

### Evaluation of the Urban Health Check PSS

A participatory evaluation framework was designed to understand the Urban Health Check PSS users’ experiences and it’s benefits to the planning agency. Based on the work of Pelzer [27] and Nielson [47], we evaluated the “utility”, and “usefulness” of the health impact PSS. The concept of “utility” was concerned with whether the PSS technology supported the planning tasks and activities of the professionals? [47, 48]. The ‘usefulness” referred to the perceived advantage of using the Urban Health Check PSS over current practices, focussing on its ability to provide better or additional insight into the nature of the planning task and whether it improved users knowledge and understanding of the potential health benefits.

**Fig. 3:**
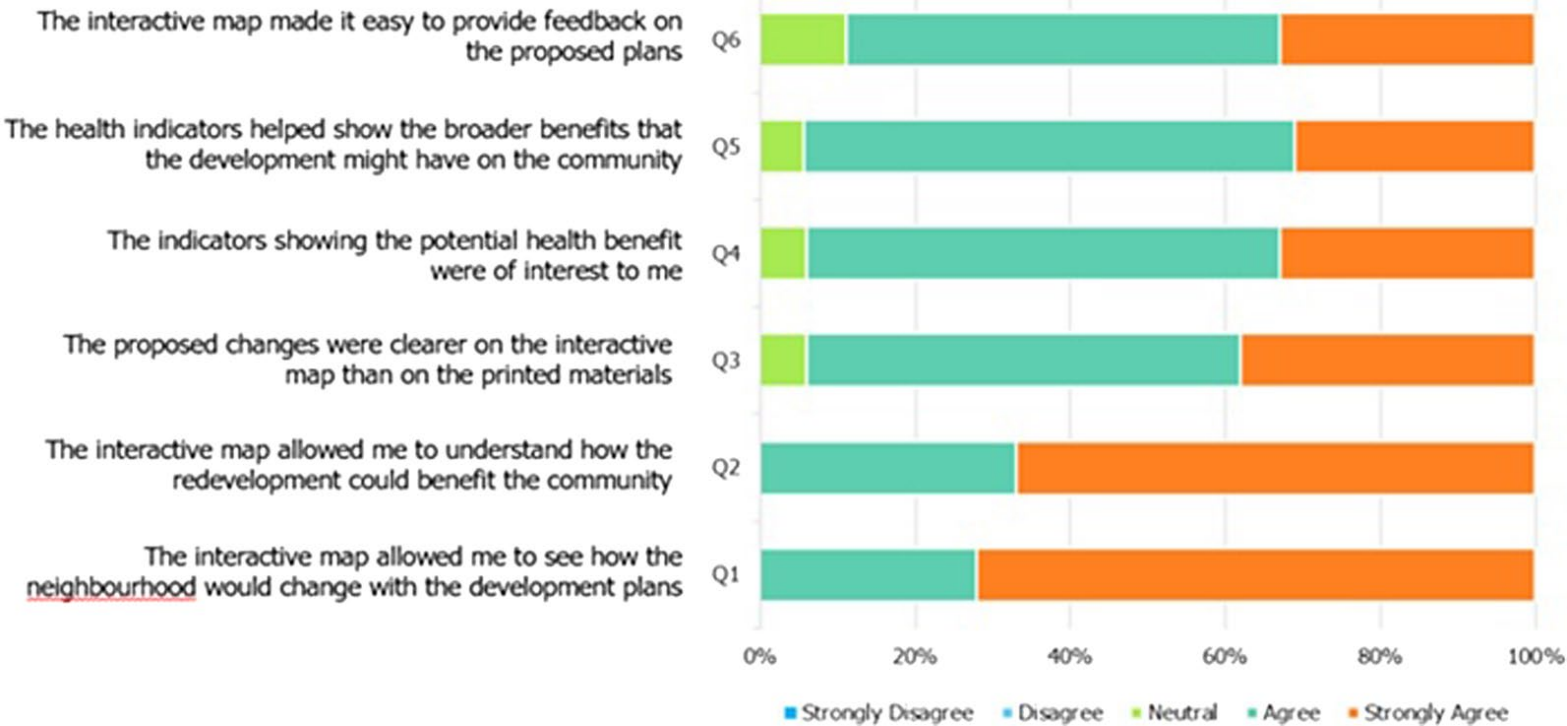
Survey results from community members

Community survey: A short survey was administered to all community members who engaged with the Urban Health Check whilst attending the community consultation event. We measured the perceived usability and usefulness of the PSS with six statements (based on [27, 48, 49] using a five-point Likert scale with higher scores indicating greater agreement with the statement. These statements related to transparency, user-friendliness and interactivity, level of detail and data quality, reliability, and communicative value (Fig. 3). Surveys were completed by 18 of the 28 participants (64 %) who engaged with the PSS. Responses indicated that the PSS helped raise awareness and understanding of the potential benefits and health impacts of the proposed redevelopment. Community feedback against the evaluation criteria found the tool was more user friendly, interactive, flexible, and provided better quality data and a level of detail and visualisation unrivalled by previous engagement methods.

Focus group workshops-planning professionals: A focus group was held with six staff from the project team, including representatives from the DevelopmentWA project (n = 2) and community consultation (n = 2) teams and the planning consultant (n = 1), to reflect on how the PSS assisted (or hindered) them in the design and consultation processes. Six 45-minute interactive demonstration workshops were then held with six Development WA staff at each (n = 36 participants) to solicit feedback on the usability and benefits and potential future applications of the Urban Health Check to the organisation and its working practices and design solutions.

- Design review: There was a general agreement that the use of the Urban Health Check PSS could support a case for greater emphasis on health and well-being in planning projects. Moreover, there was a consistent view that strength was the ability to customise the Urban Health Check PSS by adding indicators to assess other health behaviours or outcomes and environmental outcomes that also impact health and sustainability (i.e. air quality, temperature/urban heat islands). The metrics’ calculation allowed the planning team and design consultants to compare the different design options and identify their impact on the surrounding community and potential health impacts. These helped the team discuss and evaluate alternatives, trade-offs and compromises to optimise the subsequent design concept. Another perceived strength of the tool was reviewing the design concepts or proposals plans throughout and at different stages of their conceptualisation and development. The workshop participants felt it provided efficiencies as unworkable ideas could be rapidly tested and eliminated to allow for more advanced solutions to be identified.
- Community consultation and engagement: The feedback and reflection from the planning staff in attendance at the community consultation event were positive for the Urban Health Check PSS. There was consensus that the PSS allowed community members to explore the impact and health benefits of the proposed design concept. The immediate visual feedback on community members ideas sketched into the system stimulated conversation and discussion with DevelopmentWA staff, the design team and planning consultants around the trade-offs and difficulties in balancing a diverse number of factors concerning the site’s redevelopment.

## Discussion

The intersection of planning support science and spatial analytics, combined with health research, is offering new possibilities for data-driven approaches to urban planning and for the translation of health evidence to equip designers and planners with multidisciplinary, science-based information to make better, evidence-informed decisions that positively influence the design and planning of urban areas [50, 51].This paper examined the potential role and previous applications of planning support systems (PSS) and software to provide a science-based health impact assessment to communities’ planning and design through the integration and application of empirical health evidence.

Despite their promise, and potential, PSS have not become widely used in planning practice. Lessons are needed on how to effectively develop and apply PSS. This lack of experience hampers the further improvement and evolution of PSS technologies and their application [52]. As such, the paper also presents. a health-impact PSS (the “Urban Health Check”) that was trialled with a state government land development agency, planning consultants and community members on a real-world redevelopment situation with the specific aim of evaluating the usefulness and benefits of integrating a health impact PSS into the planning process in providing. planning professionals and policymakers with information to understand how different neighbourhood design approaches might impact community health and well-being and thus ‘bridge the gap’ between urban planning and public health. This is important knowledge to fill an important gap in PSS research, and to inform the future evolution and development of health impact PSS.

### Current health impact PSS

Our review identified two studies that had developed and deployed health-impact focused PSS: (1) The National Public Health Assessment Model (N-PHAM) [39] and (2) the Walkability Planning Support System [41]. The health impact components of both PSS were driven/underpinned by rigorous empirical analyses and modelling to identify coefficients describing the strength of association between multiple built environmental features and health outcomes of interest. Thirteen planning support system (PSS) products that have evolved from prototypes to fully developed professional software packages or products were identified addressing land use transportation planning and environmental impact analyses. Of these, only three incorporated a health impact analysis component. We identified one off-the-shelf proprietary software product—CommunityViz Scenario 360 [36] that would allow users to build a health impact analysis. This dynamic plug-in for ESRI’s ArcGIS enables the programming of custom models that link the spatial features to outcomes of interest (i.e., health behaviours). As proprietary products, both Envision tomorrow and Urban Footprint use their US-based models and background land use data to calculate health indicators, limiting their applicability. The models underpinning the Urban Footprint and Envision Tomorrow were developed from the same The National Public Health Assessment Model of Schoner et al., (2018) [39]. As a plug-in to ESRI’s ArcGIS software, CommunityViz provides more flexibility as it also offers the possibility for the user to define formulas for customised health and spatial indicators in different study sites and locations and scales.

Similar to the attempts of Boulange et al. [41], we have demonstrated that empirical models of the relationship between the built environment and health-related outcomes can be accommodated within CommunityViz to create a bespoke, interactive, analytical tool to test scenarios of changes in the built environment. This moves beyond conventional research translation approaches from the public health field through its ability to introduce an academic evidence-base to the world of practitioners and decision-makers who rarely use evidence (or PSS) in practice [13, 33] and to provide decision-makers with the opportunity to trial different scenarios of planned or potential interventions and to assess them against health-oriented indicators. Because the Urban Health Check PSS was built on a customisable system (CommunityViz), the proposed methodology can be applied to construct further PSS applicable to other projects and contexts and locations.

### Assessing the value of the urban health check PSS

Previous reviews into the use of PSS in the planning profession have concluded that PSS research still has to prove its added value to planning practice [28] through empirical research that moves away from experimental case studies towards real-world planning problems [30–33]. Moreover, PSS performance measures have shifted from a focus on their technical functionalities to their performance concerning their usefulness for assisting specific planning tasks [25]. This shift is highly relevant in thinking about the use of health impact PSS for the translation and application of health evidence into planning practices and design outcomes. [41]

Traditionally PSS have been developed by academic researchers for planning professionals [49]. More recently, Russo et al. [33] have emphasised the need for a co-design approach to the development of PPS that includes the participation of the planning professionals in the PSS design team to address the common problem of mismatch between PSS functionality with end-user expectations [31, 53]. Similarly, Biderman and Swiatek [51] stress the need for and added value of collaboration between politics and knowledge institutes (i.e., ‘evidence-based public policymaking in partnership with research institutes and universities’, p. 267). In the same vein, Luque-Martın and Pfeffer [54] promote ‘bridging academia and practice’ together and advocate that ‘academics and practitioners should join efforts in testing and researching the development and application of the different PSS components as an effective way to realise the desired outcomes of planning practices’. Indeed, Dias et al. [55] describe the use of interactive PSS in participatory planning processes as a promising way to bridge the gap between architects and urban designers’ creative design process and the more analytical process of planners. The Urban Health Check PSS was developed in collaboration with our industry partner to ensure it matched the planning tasks required. The spatial and health indicators were chosen to address the project’s specific design principles and community concerns, thus ensuring their relevance and fit for purpose.

The development of PSS has traditionally been focused on supporting individual decision making [20]. However, more recent approaches have seen the application of these to support group decision making as part of dynamic workshops or processes in land use planning, to engage a range of key actors and stakeholders interactively and to stimulate cooperation and improve knowledge exchange among decision-makers [20]. PSS have previously been identified as useful for planning practice by helping the public to express their needs, promoting interpersonal dialogue and debate and producing information in a form that can be understood and used by the ‘non-specialists’ [56]. However, community members engaging with our Urban Health Check PSS were reluctant to engage “hands on” with the PSS, preferring to let the facilitator sketch their ideas in the system. This might reflect the unfamiliar nature of GIS and PSS for non-professional and those competent in GIS skills.

Whilst the results of our focus groups indicated a positive response to the outputs of health impact PSS, there remains a number of obstacles to acceptance and use of PSS (in general) that could hamper the uptake and use of health impact PSS. Previous work by Vonk and Geertman [52] has identified the main bottlenecks concerning user acceptance were a lack of awareness concerning the existence and potential of PSS in planning practice, a lack of experience in using PSS and knowledge of and competency using spatial data and geographical information systems, and a general lack of intention to use PSS by the actors in the planning community. As a plug-in to ESRI’s ArcGIS, community viz. users need a degree of competency and experience using ArcGIS to create the spatial inputs and program the underling models and formulas for a PSS. The Urban Footprint and Envision Tomorrow platforms also require a level of familiarity with spatial data and GIS-based skills for the application and interpretation of the available tools and analyses – which may hamper their widespread uptake and use to date.

Further studies and evaluations, such as the one we present here, are needed to generate a better understanding of the factors influencing PSS’s actual usefulness in practice. This will enable effective solutions to the current implementation gap of PSS to be identified [18, 23, 30, 58] and improve the evolution of dedicated health impact PSS for healthy cities—helping to bridge the current research-practice gap between public health and urban planning.

Using an evaluation framework adopted from Pelzer [27, 48] that addresses the issues of usability and usefulness, we assessed the success of the Urban Health Check PSS in assisting with two distinct planning tasks identified by our industry partner and its ability to communicate health impacts of planning and design scenarios. Evaluation results indicated the PPS helped in four key areas:

1. Visualisation: the tool allowed stakeholders to see how the neighbourhood would change in response to a proposed plan.
2. Understanding: the tool helped stakeholders understand how the plan could benefit the community and demonstrate the complexity of balancing several desirable outcomes within a concept plan.
3. Health impact: the health indicators improved staff understanding of planning and design decisions that positively impact health outcomes and allowed for the communication and illustration of the broader health-related benefits to the community.
4. Engagement: the tool made it easier for community members to provide direct feedback and see the immediate implications of amendments to a proposed plan.

### Limitations of health impact PSS

The walkability PSS by Boulange et al., and our Urban Health check PSS were limited to estimating a single health (behaviour) outcome—the likelihood of walking for transport. The N-PHAM and Urban Footprint and Envision Tomorrow models included a number of different health behaviours, including walking, cycling, walking to school as well as BMI, blood pressure, diabetes and a measure of poor population health [34]. The predictive health modes underpinning the N-PHAM, Urban Footprint and Envision Tomorrow PSS and the walkability PSS were derived from large-scale population health and travel surveys and multivariate regression analyses that accounted for all of the modelled built environment variables simultaneously as well as applicable covariates including gender and age. The Urban Footprint and Envision Tomorrow models also stratify the results by gender—reflecting important and known associations of age with the health outcomes of interest.

The linear modelling approaches used in all identify PSS for the health impact indicator has limitations because it does not consider the dynamic environment in which walking is undertaken. Alternative statistical models should be tested to simulate better the complex pathways through which neighbourhoods’ design influences walking.

The health impact PSS software and studies identified have limitations associated with the underlying health impact models. A limited set of built environmental variables have been included. Whilst different across the various software and tools, the models typically included macro-level Urban Design and built environmental features associated with walking, cycling and physical activity outcomes such as landuse mix, access to public transport, retail and other daily use destinations, schools, green space, amount of green space, dwelling mix and residential densities.

In our case study of the Urban Health Check and the walkability PSS model developed by Boulange et al. (ref) did not make interventions to the street network. It is unclear from whether the N-PHAM model that underpins the Urban Footprint and Envision Tomorrow modules allow and account for changes to the street connectivity. Given, connectivity is an important design feature associated with walking outcomes, the ability to modify the street network whilst allowing real time dynamic updates warrant further investigations in future health impact PSS. Other micro-level design factors are also important in influencing walking behaviours, for example, the presence of footpaths, trees and shade or traffic volumes, but these have not been tested and included in the statistical models to date. Moreover, other variables such as safety conditions are important factors determining walkability outcomes and have important impacts on the health behaviours modelled in the identified PSS. However, the health identified health impact PSS have not included safety factors in their underlying models.

The health outcomes included in the identified PSS have focussed on physical health behaviours—such as walking, cycling and physical activity, and physical health outcomes, such as blood pressure. None of the health impact PSS identified included estimates for mental health or social health outcomes—such as sense of community, that have been extensively studied with built environments. Future health impact PSS could look to include such outcomes. Given the limited resources, economic estimates can help make public health policy decisions by quantifying the costs and benefits of different alternatives [59]. The Urban Footprint and Envission Tomorrow software include fiscal modules monetising the health impacts of design scenarios that will further assist in land use and transportation decisions and research translation. PSS also present an opportunity to explicitly communicate information about the potential health impacts of urban planning policies using spatial indicators that reflect local planning policies and are essential for research translation [12–14]. Moreover, the identified health impact PSS tools and software were developed for, and applied in, a limited number of ocations in Canada, the US and Australia. More work is needed to apply these health impact PSS to a variety of spatial contexts, locations and scales of the built environment.

Lastly, the health impact PSS software and studies identified here, as well as our pilot Urban Health Check, assess simulated alternative urban design scenarios or futures. However, the actual built form that eventuates may be markedly different. Despite this, health impact PSS have an important role to play in ensuring health is considered in the design and planning phases. More studies, that can evaluate and demonstrate the benefits of health impact PSS in educating policy makers and planners of the importance and impact of their decisions on the health of the communities they are planning for is an essential first step to ensure policies and plans include the design features needed for optimal on-ground outcomes to be achieved.

## Conclusions

We conducted a unique review into the use of PSS to include robust, empirically-based models to assess health impacts of planning and urban design concepts or scenarios for the translation and application of health research evidence. Despite some limitations associated with the health modelling approach and capabilities, we propose that the use of health-impact PSS could be transformative for the translation and application of health evidence into planning policy and practice. Furthermore, it represents a significant paradigm shift within the industry, providing, for the first time, those responsible for the policy and practice of designing and creating our communities with access to quantifiable, evidence-based information about how their decisions might impact community health. This shift could stimulate strategic decisions and prioritise design solutions tailored to optimising communities’ health outcomes and ultimately produce better, healthier on-ground communities. We provide several recommendations on how such PSS could be adopted to assist with the integration of empirical health research and empirical evidence in practice:

- Empowering planning professionals: Provides those responsible for the policy and practice of designing and creating our communities with access to quantifiable, evidence-based information about how their decisions might impact community health & well-being, testing scenarios and getting answers in real-time, in a form suited to their existing working processes & practices.
- Educate elected members: on the potential health impacts of their decisions—providing them with the knowledge, evidence and confidence to support their decision making, often in the face of community resistance to change.
- Enable planners to better communicate and engage the community: in the consultation processes and improve community awareness and understanding of proposed design and redevelopment proposals’ potential health benefits. The application of an evidence-based health-impact planning support system might help de-politicise the issue of, and address community uncertainty, debate and NIMBYism (not in my backyard) around infill and densification projects. The visualisation of the health data makes it more accessible, the impacts transparent, and the conversations evidence-based, allowing for a more rational conversation.
- Equip the next generation of young and future planners: whose education, training and professional development have not traditionally incorporated a health promotion focus with the knowledge and skills to design the health-promoting communities of the future. Specifically, training future planners to use PSS tools throughout their university studies would assist in the confidence of planners in using these tools in practice, as reported by Russo et al. (2018) [33].

We recommend that those involved in the development, use and research of health-impact PSS employ these lessons to improve the quality of PSS and their practical application and evaluate the use of health evidence. In so doing, PSS may progress to becoming the valuable tools for enhancing the role of health evidence and knowledge in planning, thereby enabling and facilitating more evidence-based planning [32, 33] and bridging the gap between public health research and planning policy and practice. However, as these health impact PSS are developed, it is imperative that evaluations and documentation of the applications to real planning applications are undertaken to ensure lessons are learnt that will inform the ongoing development and evolution of health impact PSS to ensure they are useful to those they are aimed to assist—thereby ensuring the research-practice gap will continue to be bridged.

## Data Availability

Not applicable.

## Abbreviations

PSS: Planning support systems
GIS: Geographical information systems
